# Prevalence of Prediabetes and Type-2 Diabetes Mellitus in Cricket Players: Multi-Cricket Clubs Cross Sectional Study

**DOI:** 10.12669/pjms.37.4.4128

**Published:** 2021

**Authors:** Sultan Ayoub Meo, Abdulelah Adnan Abukhalaf, Ali Abdullah Alomar, Waqas Sami, Anusha Sultan Meo

**Affiliations:** 1 Sultan Ayoub Meo, Department of Physiology, College of Medicine, King Saud University, Riyadh, Saudi Arabia; 2 Abdulelah Adnan Abukhalaf, Department of Physiology, College of Medicine, King Saud University, Riyadh, Saudi Arabia; 3 Ali Abdullah Alomar, Department of Physiology, College of Medicine, King Saud University, Riyadh, Saudi Arabia; 4 Waqas Sami, Department of Public Health, University of Health Sciences, Lahore, Pakistan; 5 Anusha Sultan Meo, Army Medical College, National University of Medical Sciences, Rawalpindi, Pakistan

**Keywords:** Cricket, Sport, Prevalence, Diabetes Mellitus

## Abstract

**Background & Objectives:**

Sports activities are highly beneficial for improving the human health and reducing the risk of diseases. This cross sectional study aimed to investigate the prevalence of prediabetes and Type-2 diabetes mellitus in cricket players compared to population based non-elite athlete control subjects.

**Methods:**

The present matched cross sectional study was conducted in the Department of Physiology, College of Medicine, King Saud University, Riyadh, Saudi Arabia during the period October 2019 to February 2020. Initially, 700 volunteer males, (300) cricket players and (400) population based non-elite athlete control subjects were interviewed. After socio-demographic and medical history, (200) nonsmoker cricket players and (300) nonsmoker control subjects were recruited. The age of cricket players was 34 (32-37) years, weight 81 (76-84) kg, height 1.79 (1.74-1.84) meters, and body mass index (BMI) was 25.09 (23.66-26.76) kg/m^2^. The cricket players have been playing cricket for 4 (3-4) hours per day; 3.50 (3-4) days per week; for the total period of 24 (12-36) months. American Diabetes Association (ADA) based criteria on Glycated Hemoglobin (HbA1c) was used to investigate the prediabetes and Type-2 diabetes mellitus.

**Results:**

In cricket players, the prevalence of prediabetes was 23 (11.5%) and Type-2 diabetes mellitus (T2DM) was 7 (3.5%) compared to population based matched non-elite athlete control subjects the prediabetes was 73 (24.34%) and T2DM was 63 (21.1%) (p=0.001). Among cricket players, there was a 6-folds decrease in T2DM compared to control subjects.

**Conclusions:**

The cricket sports activities decrease the prevalence of prediabetes and Type-2 diabetes mellitus (T2DM) among the cricket players compared to population based matched non-elite athlete control subjects. The study findings demonstrate the urgent need for promoting sports activities, more cricket grounds as a physiological preventive strategy against the global growing diabetes epidemic.

## INTRODUCTION

Diabetes mellitus is a major global challenge, allied with substantial morbidity, mortality and huge economic burden on the healthcare system.^1^ Despite amazing developments in medical sciences, it is still an incurable life-long disease.^2^ The recent global prevalence of diabetes mellitus is 463 million; 374 million people are suffering from impaired glucose tolerance whereas 232 million people are unaware of the fact that they are suffering from the disease. Diabetes caused 4.2 million deaths in year 2019, 11666 people per day and 8.10 people per minute. Moreover, the world health expenditure on diabetes is US$ 760 billion.^3^

Currently, diabetes mellitus has a high rank on the international health agenda due to being a deathtrap to human health and worldwide economies.^1^,^4^ Globally, many states have developed policies to arbitrate on risk factors, such as lifestyle, smoking, diet, physical activity, to reduce the prevalence of debilitating diseases.^5^ Lack of physical activity, unhealthy diet, and sedentary lifestyle account for an obesity and diabetes mellitus.^6^

The sports allied physical activities improve the health and capacity of the individual’s and performance. The sports activities including cricket sport has been acknowledged as a potential health promotion strategy to reduce the sedentary behavior. Sport improves endurance capacity and has a positive influence on cardiovascular and metabolic health.^7^ The sport-based interventions in sedentary people can achieve primary preventive effects and far-reaching improvements in health.^8^ In recent years, the evidence for the health benefits of sport showed that it improves aerobic fitness, muscular performance, metabolic and cardiovascular function and reduces adiposity.^9^ However, literature is extremely lacking to establish an association between playing cricket and prevalence of Type-2 diabetes mellitus (T2DM). This study aim was to investigate the prevalence of prediabetes and T2DM in cricket players compared to population based non-elite athlete matched control subjects.

## METHODS

### Study design and settings

The present matched cross sectional study was conducted in the Department of Physiology, College of Medicine, King Saud University, Riyadh, Saudi Arabia during the period Oct. 2019 to Feb. 2020.

### Study participants

In this study, various schools, colleges, universities, and small- and large-scale cricket sports grounds were visited and information about cricket players was gathered. Initially 300 volunteer male cricket players, and (400) population based non-elite athlete control subjects were interviewed. After socio-demographic, medical history and examination, a total of 500, (200) nonsmoker cricket players and (300) nonsmoker control subjects were recruited. The age of cricket players was 34 (32-37) years, weight 81 (76-84) kg, height 1.79 (1.74-1.84) meters, and Body mass index (BMI) was 25.09 (23.66-26.76) kg/m.^2^ The cricket players have been playing cricket for 4 (3-4) hours per day, 3.50 (3-4) days, and a total period of 24 (12-36) months (Table-I). It was ensured that these players were involved in cricket sport only and no other sports allied activities such as volleyball, badminton, football, hockey, swimming etc. Moreover, these cricket players were not involved in working exposure to any industries such as cement, coal, cotton, oil, flour, factories as these industries generate pollution, and pollution increases the prevalence of diabetes mellitus.^2^,^10^,^11^

**Table-I:** Comparison of Anthropometric, clinical, and other parameters among cricket players and matched controls.

Parameters	Cricket Players Median (25^th^-75^th^) quartile	Controls Median (25^th^-75^th^) quartile	p-value
Age (year)	34 (32-37)	33 (32-37)	0.803
Height (m)	1.79 (1.74-1.84)	1.70 (1.66-1.75)	0.841
Weight (kg)	81 (76-84)	76 (72-81)	0.451
BMI (kg/m^2^)	25.09 (23.66-26.76)	26.47 (25.35-27.60)	<0.001*
HbA1c	5.30 (5.10-5.50)	5.60 (5.20-6.37)	<0.001*

* Statistically significant at 5% level of significance.

Similarly, for the control group, various schools, colleges, universities were visited and initially, 500 population based non-elite athlete control subjects were interviewed. After socio-demographic and medical history examination, 300 control subjects were selected from schools and universities clerical staff, technicians, research assistants. The median age for the non-elite athlete control subjects were 33 (32-37) years, weight 76 (72-81) kg, height 1.70 (1.66-1.75) meters, and Body Mass Index was 26.47 (25.35-27.60) (kg/m2). It was warranted that these control subjects were not involved in sports activities such as football, volleyball, badminton, hockey, swimming etc. Moreover, these control subjects were not involved in working exposure to any industries such as cement, coal, cotton, oil, flour, factories as these industries generate pollution, and pollution increases the prevalence of diabetes mellitus.^2^,^10^, ^11^ A verbal consent was taken from the participants.

### Clinical history and Socio-demographic characteristics

**Fig.1:**
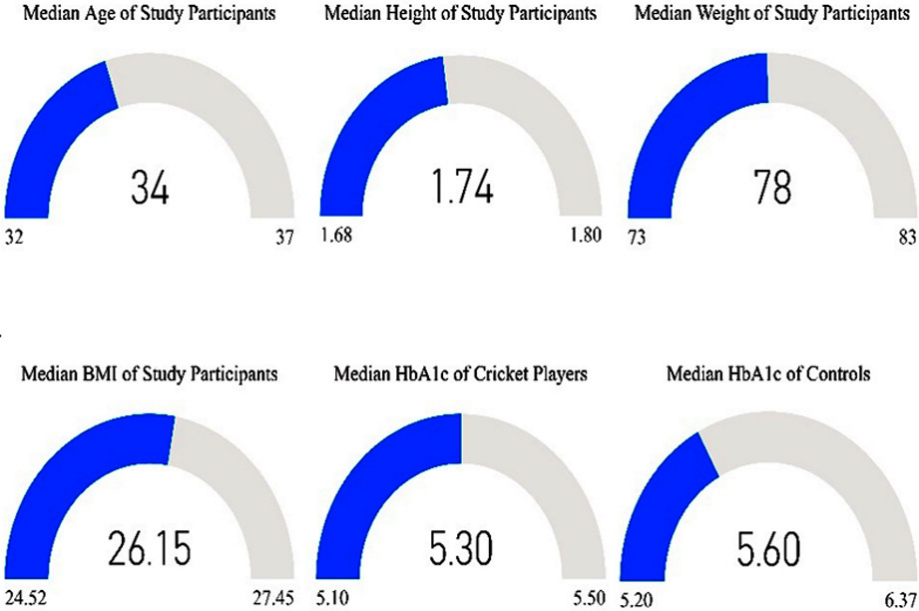
Median (25^th^ -75^th^) quartiles for various study parameters.

Two co-investigators interviewed 300 volunteer male cricket players and (400) population based non-elite athlete control subjects were interviewed. A detailed sociodemographic and medical history was obtained. The information about age, gender, height, weight, BMI, duration of playing cricket, demographic characteristics, lifestyle, diet habit, physical activities and other health-related information was collected by the use of a questionnaire. The “socio-demographic characteristics including residential address, living conditions, education level, marital status, monthly income, lifestyle information, and smoking were recorded. Other health-related evidence including family history of diabetes mellitus was also taken”. Both groups were matched for, age, weight, BMI, socioeconomic, and dietary habits. After demographic, medical history and examination, finally a total of 500, (200) nonsmoker cricket players and (300) nonsmoker control subjects were recruited (Fig.1).

### Exclusion Criteria

Participants with a known history of “anemia, blood diseases, blood transfusion, asthma, diabetes mellitus and malignancy were excluded from the study. Subjects who smoke traditional or electronic cigarette, or shisha were also excluded.”^12^ It was ensured that the cricket sports players were only playing cricket and control group participants were population based non-elite athlete subjects. The participants with a current or “previous history of an employment in any industrial plant which produces dust or fumes such as plastic, cement, coal, cotton and flour factories were also not included in the study”.^10^,^11^

### Measurements of Glycated hemoglobin (HbA1c)

The cricket players and control subjects were allotted an identification number, and a para-medical staff was assigned to measure the HbA1c. The “HbA1c was measured using the Clover A1c system (Inforpia, Kyunggi, Korea), an automated boronate affinity assay for the determination of the percentage of HbA1c % in the whole body’s blood.^13^ American Diabetes Association (ADA)^14^based criteria on glycated hemoglobin (HbA1c) were used to diagnose the diabetes mellitus”. Subjects with “HbA1c less than 5.7% were considered as non diabetics; HbA1c 5.7%-6.4% as prediabetics; and subjects with HbA1c more than 6.4% were considered diabetics.”^14^ HbA1c is a reliable indicator of glycemic measurements for the diagnosis of diabetes mellitus.^14^,^15^

### Ethics statement

This study was executed in harmony with the “Declaration of Helsinki”, and the protocol was approved by the “Ethics Committee, College of Medicine Research Centre, King Saud University (E-19-4494)” (Ref: 20/0106/IRB, Dated: 18-02-2020).

### Statistical Analysis

The data was analyzed using SPSS 26.0 (IBM Corp., Armonk, N.Y., USA) and Microsoft Power BI, 2020. Normality of the data was checked by one-sample Kolmogorov-Smirnov test. Median (25^th^-75^th^ quartiles) are reported for non-normally distributed quantitative variables (age, height, weight, BMI, HbA1c, playing cricket hours, days, and months). Levene’s test was applied to check the variability in the anthropometric measures between the study groups. Mann-Whitney U test was applied to compare various parameters among study groups. A two-sample proportion test was also applied to observe the association between qualitative variables. Binary logistic regression was applied to observe the log-odds between HbA1c and study groups controlling for anthropometric measures. An α=0.05 was considered statistically significant. Spearman Rho Correlation was applied to assess the relationship between HbA1c, cricket playing hours, days, and months at 1% level of significance.

## RESULTS

The anthropometric, clinical data and other variables were not normally distributed. A total of 500 participants were enrolled in the study of which 200 (40%) were cricket players and 300 (60%) were controls. Both the study groups were matched for age, height, and weight (Table-I). For the whole study sample, the median (25^th^ – 75^th^ quartile) for age was 34 (32-37) years, weight 78 (73.25-83) kg, height 1.73 (1.68-1.80) meters, BMI 26.14 (24.52-27.45) kg/m^2^ and HbA1c 5.30 (5.10-5.50)% (Fig.1). The median playing cricket hours were 4 (3-4), days 3.50 (3-4) and months 24 (12-36). Cricket players had a significantly lower median HbA1c and BMI level than the controls (p<0.001) respectively, however no significant difference was observed in the age, height, and weight between the two study groups (p>0.05) respectively, Table-I & Fig.1.

**Fig.2:**
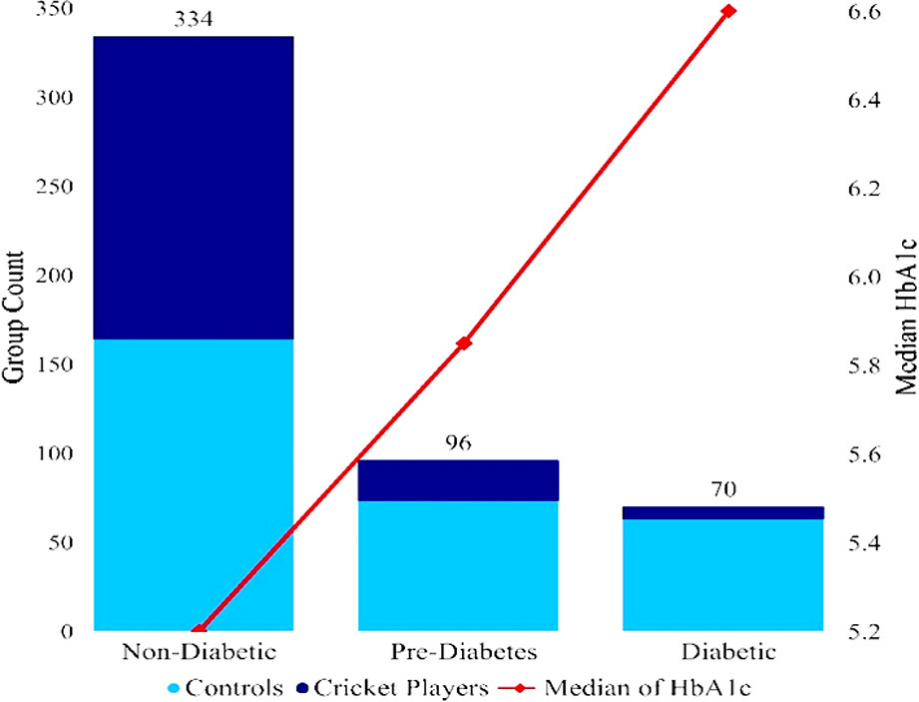
Non-diabetes, pre-diabetes and T2DM among cricket players and matched controls in relation with median HbA1c level

**Figure 3:**
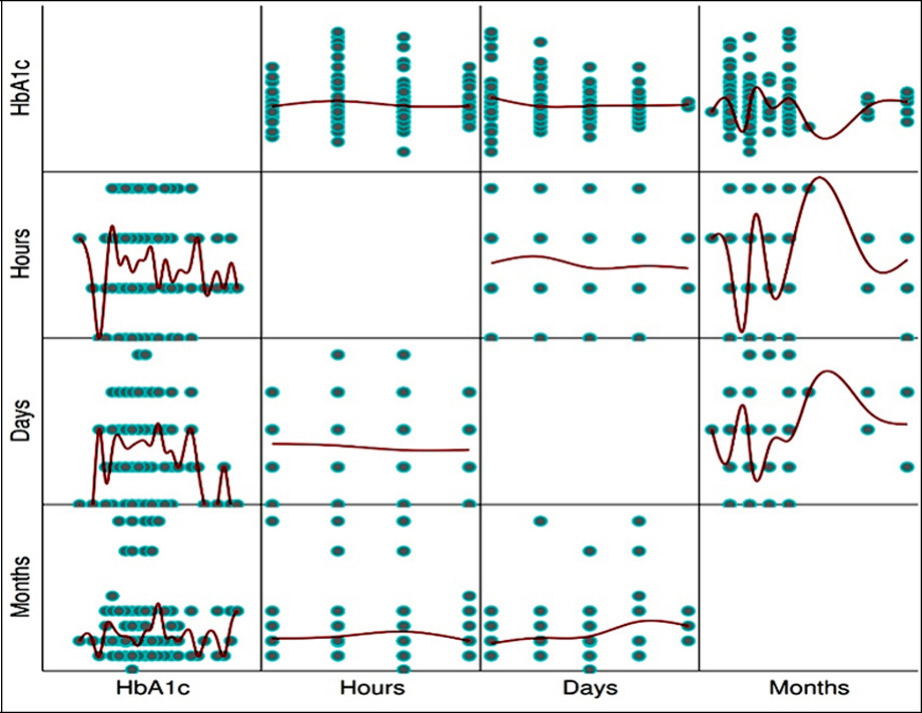
Scatter -Plot Matrix, HbA1c, playing cricket hours, days, and months.

Among cricket players the prevalence of pre-diabetes 23 (11.5%) was significantly lower than their matched pre-diabetic controls 73 (24.34%) p<0.001, similarly cricket players had a significantly lower T2DM 7 (3.5%) when compared with the matched controls having T2DM 63 (21%) p<0.001. Moreover, the proportion of non-diabetes was again significantly lower in cricket players 170 (85%) than their matched controls 164 (54.66%) p<0.001, Table-II and Fig.2. Binary logistic regression was also applied, the dependent variable was (cricket players and controls), it was used to predict the HbA1c after controlling for age, height, weight, and BMI. The model chi-square value was significant at 1% level of significance confirming that the fitted model was appropriate. The overall classification accuracy obtained was 75.6%. Playing cricket showed a protective effect from developing T2DM, further results showed that playing cricket can significantly reduce the HbA1c level by 73.7% [Adjusted Odds Ratio= 0.263; 95% CI=0.166–0.418]. However, among cricketers, no significant correlation was observed between HbA1c level, number of crickets playing hours (ρ= -0.032, p=0.656), days (ρ=-0.028, p=0.692) and months (ρ=0.018, p=0.797) Fig.3.

**Table-II:** Association of non-diabetes, pre-diabetes and T2DM among cricket players and matched controls.

Parameters	Controls N (%)	Cricket Players N (%)	Total	p-value
Non-Diabetic (HbA1c <5.7%)	164 (54.66)	170 (85.0)	334	0.696
Pre-Diabetic (HbA1c 5.7%-6.4%)	73 (24.34)	23 (11.5)	96	<0.001*
Diabetic (>6.4%)	63 (21.0)	7 (3.5)	70	<0.001*

* Statistically significant at 5% level of significance

HbA1c values are classified as per American Diabetes Association Guidelines.^14^

## DISCUSSION

Cricket is a highly popular sport played by both gender, all age groups, and have many health benefits. This is the first study to investigate the prevalence of prediabetes and T2DM among cricket players compared to population based non-elite athlete control subjects. In this study we found that the prevalence of T2DM in cricket players was 3.5% compared to their matched control group (21.0%) with age group 32-37 years. These findings suggest that T2DM is almost 6 folds less in cricket players compared to their age, weight, BMI matched control subjects. The findings are interesting from the perspective that the prevalence of T2DM was significantly low in cricket players compared to non-elite athlete control subjects in a region where T2DM is highest across the globe.^16^

The cricket sport is a highly popular game with various recreational activities. The cricket is a high-intensity sport with a positive impact on glucose metabolism.^17^ Nieuwoudt et al.^18^ reported that after six weeks high-intensity functional training (HIFT) among adults with T2DM, participants showed significant improvements in beta-cell function while decreasing the body fat and preserving lean mass. In another study, Fealy et al.^19^ reported an increased insulin sensitivity after 6-week HIFT training. It has also been reported that sports allied activities increases insulin sensitivity, positively influencing glycemic control, and potentially providing better tools for the prevention of T2DM.^20^

Lao et al.^21^ evaluated the effect of habitual leisure-time physical activity (LTPA) on T2DM incidence. The authors reported that high levels of LTPA are associated with a lower risk of diabetes. Sarmento et al.^22^ reported the benefits of sport on cardiovascular, bone health, and body composition, as it increased insulin sensitivity, and had a positive impact on glycemic control and T2DM. Krustrup et al.^23^ describes the health effects of recreational female football players. The study documents that 2 × 1 h of recreational training for 12-16 weeks causes marked health improvements. The authors concluded that regular sports activities for women is an effective tool for prevention and treatment of hypertension T2DM.

Meo et al.^24^ demonstrated that, in football players the prevalence of prediabetes and T2DM was significantly low compared to control subjects The present study findings shows significant decreased prevalence of prediabetes and T2DM among cricket players compared to non-elite athlete control subjects.

The potential mechanism involved in playing cricket decreases insulin resistance and diabetes mellitus is highly interesting to understand. The epidemiological literature acknowledges the fact that sports activities decreases the insulin resistance and ultimately leads to decreased risk of T2DM. Cricket creational activities decreases oxidative stress, improves the antioxidant capabilities, glucose intake and decreases T2DM.^25^ Moreover, sports activities enhance insulin sensitivity, glucose transport into muscle cells^22^,^23^, and increase production of muscle glycogen to replace the glycogen used during the exercise. It may also exert a long-term effect on improvement in insulin sensitivity through increased fat-free mass, which increases the volume of muscle tissue into which glucose can be transported.^20^

The sports activities are beneficial for people to fight against debilitating diseases including cardio-metabolic disorders. it increases the concentration of GLUT-4 in the cell membrane and increases glucose uptake in skeletal muscles.^26^-^28^ Acute exercise increases glucose tolerance, insulin sensitivity and decrease blood glucose levels.^29^,^30^ The literature has also shown that acute and moderate-intensity endurance exercise decreases the blood glucose levels.^29^,^30^ These are the possible mechanisms in playing sports including cricket leads to decrease in prevalence of prediabetes and T2DM.

### Strengths and Limitations of the study

This is the first study to investigate the prevalence of prediabetes and T2DM in cricket players. The study exclusion criteria was well established, cigarette smokers were excluded. Both groups were matched for age, height, weight, BMI, ethnicity, and socio-economic levels to minimize the possible confounding factors. American Diabetes Association diagnosis approach was followed, Glycated Hemoglobin (HbA1c) is a reliable and valid indicator to identify an individual’s long-term mean blood glucose levels criteria was empoyed. This study could therefore be the best reference on the prevalence of prediabetes and T2DM. There are some limitations that we would like to point out, despite trying to recruit a large number of cricket players, we excluded cigarette smokers, age, weight, height and ethnicity matched criteria was employed hence we excluded a large number of participants and finally included 200 cricket players and 300 control subjects. Moreover, due to cultural limitations, we only include the male gender.

## CONCLUSIONS

Cricket allied recreational activities significantly decrease the prevalence of prediabetes and T2DM in cricket players compared to matched control subjects. The decreased prevalence was associated with the duration of cricket activities. The findings have public health implications, and supports the extension of diabetes intervention efforts. Health officials should establish more sports facilities, cricket grounds for the public to provide better sports facilities which will help in minimizing the incidence of prediabetes and T2DM. Public policies to promote sports activities have potential to improve population health and minimize the healthcare expenditure.

### Author’s Contributions:

**SAM** designed the study, applied research grant, ethics board approval, literature review, data analysis, manuscript writing and overall supervision of the project,

**AAA, AAA, ASM** literature review, data collection.

**WS** data analysis.
